# Association between advanced lung cancer inflammation index and chronic kidney disease: a cross-sectional study

**DOI:** 10.3389/fnut.2024.1430471

**Published:** 2024-10-04

**Authors:** Xiaotong Li, Qian Wang, Feng Wu, Ziyang Ye, Yafeng Li

**Affiliations:** ^1^The Nephrology Department of Shanxi Provincial People's Hospital, Shanxi Medical University, Taiyuan, China; ^2^Shanxi Provincial Key Laboratory of Kidney Disease, Taiyuan, Shanxi, China; ^3^The Third Clinical College, Shanxi University of Chinese Medicine, Taiyuan, Shanxi, China; ^4^Chronic Kidney Disease Medical and Pharmaceutical Basic Research Innovation Center of the Ministry of Education of the People's Republic of China, Taiyuan, China; ^5^Core Laboratory, Shanxi Provincial People's Hospital (Fifth Hospital), Shanxi Medical University, Taiyuan, China; ^6^Academy of Microbial Ecology, Shanxi Medical University, Taiyuan, China; ^7^Hejin Municipal People's Hospital, Hejin, China

**Keywords:** advanced lung cancer inflammation index, chronic kidney disease, nutrition, inflammation, NHANES, cross-sectional study

## Abstract

**Background:**

Chronic kidney disease (CKD) is one of the common chronic diseases, and malnutrition and inflammation play a key role in the development of CKD. The advanced lung cancer inflammation index (ALI) is a novel index of nutrition and inflammation, and its association with CKD has not yet been clarified. The aim of this study was to explore the potential association between ALI and CKD.

**Methods:**

We conducted a cross-sectional survey using data extracted from the National Health and Nutrition Examination Survey (NHANES, 2003–2018). Weighted multivariate logistic regression was used to assess the association between ALI and CKD, and smoothed curve fitting and threshold effect analyses were used to describe the nonlinear association between ALI and CKD. Subgroup analyses were performed to further assess the influence of other covariates on the relationship between ALI and CKD.

**Results:**

A total of 39,469 adult participants were included in the study, of whom 7,204 (18.25%) were diagnosed with CKD. After adjusting for multiple confounders, we found a significant negative correlation between ALI and CKD (OR = 0.93; 95%CI, 0.91–0.95; *p* < 0.0001). The risk of CKD tended to decrease with increasing quartiles of ALI. Smoothed curve fitting showed an L-shaped negative correlation between ALI and CKD. Threshold analysis showed a saturation effect of ALI at the inflection point of 55.09. Subgroup analyses and interaction tests showed that this negative association was maintained across age, sex, race, BMI, diabetes, hypertension, cardiovascular disease, and cancer subgroups (P for interaction >0.05).

**Conclusion:**

Our findings suggest a significant correlation between ALI and CKD in the US adult population. However, more large-scale prospective studies are still needed to further confirm our findings.

## 1 Introduction

Chronic kidney disease (CKD) is a common progressive disease whose prevalence is increasing globally, with current statistics increasing the number of people living with the disease to approximately 850 million worldwide (1). In addition, the development of CKD is often accompanied by increased rates of cardiovascular disease comorbidity and mortality. This not only increases the medical burden on patients, but also has a serious impact on their quality of life and mental health (2). The pathogenesis of CKD is complex and involves multiple pathways and factors. Among them, inflammation and malnutrition are risk factors for disease progression and poor prognosis in CKD (3, 4). Microinflammatory state is prevalent in CKD patients, and more than 50% of patients are accompanied by active inflammation (5, 6). Inflammation adversely affects the nutritional status by promoting protein metabolism, increasing energy consumption, and suppressing appetite, which is closely related to the prevalence of disease and mortality in CKD patients (7).

Recently, there has been increasing evidence that multiple nutrition/inflammation-related indices can serve as effective predictors of CKD. Inflammatory indices such as neutrophil-to-lymphocyte ratio (NLR) (8, 9), systemic immune-inflammatory index (SII) (10), and systemic inflammatory response index (SIRI) (11) have been identified as being associated with the development and prognosis of CKD. In addition, based on indicators assessing nutritional status, the geriatric nutritional risk index (GNRI) (12) and prognostic nutritional index (PNI) (13) are important prognostic indicators of CKD progression. The advanced lung cancer inflammation index (ALI) is a comprehensive index developed in recent years to assess the nutritional and inflammatory status of patients, which consists of body mass index (BMI), serum albumin and NLR (14, 15). Among them, serum albumin and BMI are commonly used in clinical assessment of nutritional status in CKD. As the name of the ALI suggests, it is primarily used to predict the prognosis of lung cancer patients, and lower levels of ALI were significantly associated with a higher risk of death from lung cancer (16). In addition, studies have found ALI to be associated with prognosis in a variety of inflammatory diseases, such as hypertension, diabetes, coronary heart disease, and Crohn’s disease (17–20). However, the association between ALI, a nutritional and inflammatory index, and CKD has not been elucidated.

Therefore, we conducted a population-based cross-sectional study to investigate the relationship between ALI and the prevalence of CKD among adult participants of the National Health and Nutrition Examination Survey (NHANES). The study confirmed a negative association between ALI and the likelihood of developing CKD.

## 2 Method

### 2.1 Data population sources and study

NHANES is a database and survey program widely used to assess the health and nutrition status of the U.S. population. It uses a sophisticated, multistage probability sampling design to select a sample representative of the non-hospitalized population of US residents. Participants include people of different ages, races, genders, and economic backgrounds. Data on the prevalence of chronic diseases in the population can be collected by medical staff through home-visit questionnaires and mobile laboratory assessments. All survey information was subjected to strict privacy measures prior to release, and all participants signed informed consent forms.

**Figure 1:**
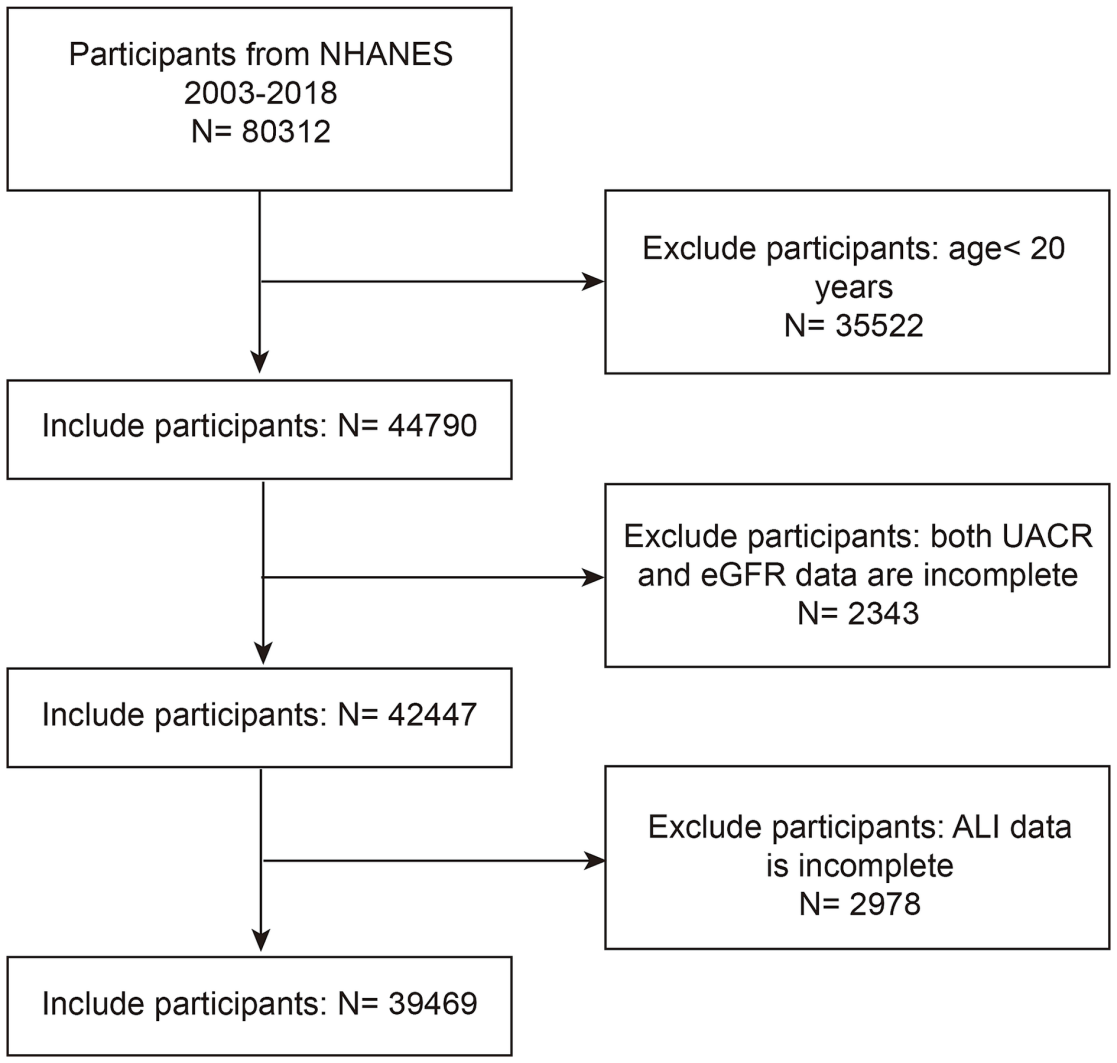
NHANES 2003–2018 participant selection flowchart. NHANES, National Health and Nutrition Examination Survey; eGFR, estimated glomerular filtration rate; UACR, urinary albumin to creatinine ratio; ALI, advanced lung cancer inflammation index.

This cross-sectional study utilized data from eight NHANES cycles spanning 2003–2004 to 2017–2018, initially including 80,312 participants. We excluded 35,522 participants under 20 years of age, 2,343 participants without available urinary albumin-to-creatinine ratio (UACR) or estimated glomerular filtration rate (eGFR) data, and 2,978 participants missing ALI data. After applying these exclusion criteria, the final analytic sample consisted of 39,469 participants (Figure 1).

### 2.2 Data collection

#### 2.2.1 Exposure variable

ALI was calculated using the formula: BMI (kg/m^2^) × serum albumin level (g/dL) / NLR. BMI = body weight in kilograms/ (height in meters)^2^ based on examination data, NLR = absolute neutrophil count/absolute lymphocyte count based on blood samples (15). Based on the level of ALI, the patients were divided into 4 groups according to their quartiles: group Q1 (2.83–44.36), group Q2 (44.37–61.90), group Q3 (61.91–83.40) and group Q4 (≥ 83.41).

#### 2.2.2 Outcome variable

eGFR was calculated using the Chronic Kidney Disease Epidemiology Collaboration (CKD-EPI) equation developed in 2009 (21). Participants with UACR ≥30 mg/g or eGFR <60 mL/min/1.73 m^2^ were diagnosed as chronic kidney disease patients (22).

#### 2.2.3 Definition of covariates

To control for the effect of confounders on study outcomes, we included covariates including age, gender, race, education level, poverty income ratio (PIR), BMI, drinking and smoking status, diabetes, hypertension, cardiovascular disease (CVD), and history of cancer. The selection of these covariates was determined based on a professional evaluation of existing studies. Race was categorized as Mexican American, other Hispanic, Non-Hispanic White, Non-Hispanic Black, and other race. Educational attainment was categorized as below high school, high school, and above high school. PIR was divided into 3 components: < 1.3, 1.3 to <3.5, and ≥ 3.5. BMI was divided into 3 groups: normal (< 25 kg/m^2^), overweight (25 to <30 kg/m^2^), and obese (≥ 30 kg/m^2^). Alcohol consumption was categorized as never drinking, former drinking, and current drinking. Smoking was categorized as never smoking, former smoking, and current smoking. The diagnosis of hypertension and diabetes was based not only on self-reported physician diagnostic information and current medication, but also on standardized laboratory test results (23, 24). CVD was defined based on self-reported access to diagnostic information and covered conditions such as heart failure, coronary heart disease, angina pectoris, myocardial infarction or stroke (15). Cancer was defined as a self-reported physician’s prior diagnosis of cancer or malignancy. Detailed information on all covariates is available at www.cdc.gov/nchs/nhanes/index.htm.

### 2.3 Statistical analyses

According to the NHANES analytical guidelines, this study fully considered the complexity of the sample design, including hierarchical structure, clustering effects, and sample weights. In this study, continuous variables were described by weighted mean (standard deviation) and analyzed using weighted linear regression for comparison. Categorical variables were described by unweighted frequencies (*n*) and weighted percentages (%) and analyzed using chi-square tests. Weighted multiple logistic regression analyses were used in this study to explore the association between ALI and CKD, including three models: model 1 did not include any covariates; model 2 adjusted for age, gender, and ethnicity; and model 3 considered the effects of all covariates. All models assessed the association between ALI and CKD by the ratio of ratios (OR) and their 95% CI. In addition, we investigated the nonlinear associations and inflection points of ALI with CKD using smoothed curve fitting and threshold effect analysis models. We also performed subgroup analyses according to age, gender, race, BMI, diabetes, hypertension, CVD, and cancer to further assess the association between ALI and CKD. All statistical analyses were completed using R studio 4.2.3 and EmpowerStats software. Differences were considered statistically significant at a *p* value of less than 0.05.

## 3 Results

### 3.1 Baseline characteristics of study participants

A total of 39,469 participants took part in this study. The mean age was 47.26 years. Among them, 48.24% were males and 51.76% were females. There were 7,204 CKD patients among all the participants, which was 18.25% of the total.

Basic information on subjects grouped by CKD status is shown in Table 1. Compared with the non-CKD population, patients with CKD tended to be older, less educated, had a lower PIR, had a greater history of prior smoking and alcohol consumption, and were more prevalent in the female and non-Hispanic populations. In addition, CKD patients had a higher prevalence of hypertension, diabetes, cardiovascular disease, and cancer, and had lower ALI levels. All differences were statistically significant (*p* < 0.05).

**Table 1:** Weighted characteristics of the study population according to the presence of CKD.

	Overall	No CKD	CKD	*p*-value
	*N* = 39,469	*N* = 32,265 (81.75%)	*N* = 7,204 (18.25%)	
Age (years)	47.26 (16.89)	44.94 (15.66)	61.10 (17.33)	<0.0001
Sex				<0.0001
Male	19,040 (48.24%)	15,865 (49.17%)	3,076 (42.70%)	
Female	20,429 (51.76%)	16,400 (50.83%)	4,128 (57.30%)	
Race				<0.0001
Mexican American	3,347 (8.48%)	2,817 (8.73%)	504 (7.00%)	
Other Hispanic	2,120 (5.37%)	1778 (5.51%)	326 (4.52%)	
Non-Hispanic White	26,839 (68.00%)	21,850 (67.72%)	5,019 (69.67%)	
Non-Hispanic Black	4,223 (10.70%)	3,381 (10.48%)	868 (12.05%)	
Other Race	2,940 (7.45%)	2,439 (7.56%)	487 (6.76%)	
Education Level				<0.0001
Below high school	6,414 (16.25%)	4,895 (15.17%)	1,633 (22.67%)	
High school	9,303 (23.57%)	7,463 (23.13%)	1887 (26.20%)	
Above high school	23,729 (60.12%)	19,888 (61.64%)	3,676 (51.02%)	
Unrecord	23 (0.06%)	19 (0.06%)	8 (0.11%)	
PIR				<0.0001
<1.3	8,308 (21.05%)	6,582 (20.40%)	1797 (24.94%)	
1.3 to <3.5	14,185 (35.94%)	11,280 (34.96%)	3,016 (41.87%)	
≥3.5	16,976 (43.01%)	14,403 (44.64%)	2,391 (33.19%)	
BMI (kg/m^2^)				<0.0001
<25	11,928 (30.22%)	10,002 (31.00%)	1839 (25.53%)	
25 to <30	13,103 (33.20%)	10,860 (33.66%)	2,195 (30.47%)	
≥30	14,438 (36.58%)	11,403 (35.34%)	3,170 (44.00%)	
Alcohol drinking status				<0.0001
Never drinker	5,139 (13.02%)	3,891 (12.06%)	1,377 (19.12%)	
Former drinker	4,282 (10.85%)	3,117 (9.66%)	1,327 (18.42%)	
Current drinker	30,044 (76.12%)	25,257 (78.28%)	4,500 (62.46%)	
Smoking status				<0.0001
Never smoker	21,515 (54.51%)	17,797 (55.16%)	3,647 (50.62%)	
Former smoker	9,760 (24.73%)	7,537 (23.36%)	2,371 (32.91%)	
Current smoker	8,194 (20.76%)	6,931 (21.48%)	1,186 (16.47%)	
Hypertension				<0.0001
Yes	12,362 (31.32%)	8,618 (26.71%)	4,242 (58.88%)	
No	27,056 (68.55%)	23,612 (73.18%)	2,945 (40.88%)	
Unrecord	51 (0.13%)	35 (0.11%)	17 (0.24%)	
Diabetes				<0.0001
Yes	4,578 (11.60%)	2,697 (8.36%)	2,158 (29.96%)	
No	34,891 (88.40%)	29,568 (91.64%)	5,046 (70.04%)	
CVD				<0.0001
Yes	3,414 (8.65%)	1939 (6.01%)	1767 (24.53%)	
No	36,055 (91.35%)	30,326 (93.99%)	543 (75.47%)	
Cancer				<0.0001
Yes	3,801 (9.63%)	2,662 (8.25%)	1,290 (17.90%)	
No	35,625 (90.26%)	29,577 (91.67%)	5,896 (81.84%)	
Unrecord	43 (0.11%)	26 (0.08%)	19 (0.26%)	
UACR (mg/g)	31.43 (13.16)	7.69 (1.36)	175.04 (31.21)	<0.0001
eGFR (mL/min/1.73m^2^)	94.04 (22.16)	97.74 (18.40)	71.90 (28.81)	<0.0001
ALI	65.96 (34.35)	66.91 (27.02)	60.27 (39.20)	<0.0001

CKD, chronic kidney disease; PIR, poverty income ratio; BMI, body mass index; CVD, cardiovascular disease; UACR, urinary albumin to creatinine ratio; eGFR, estimated glomerular filtration rate; ALI, advanced lung cancer inflammation index. Continuous variables were described by weighted mean (standard deviation), categorical variables were described by unweighted frequencies (*n*) and weighted percentages (%).

The clinical characteristics of the participants according to quartiles of ALI are shown in Table 2. eGFR levels were higher, and the prevalence of albuminuria and CKD was lower in participants in the higher ALI quartiles. In addition, those in the lower ALI quartiles were more likely to have diabetes, cardiovascular disease, and cancer than those in the other categories. All differences were statistically significant (*p* < 0.05).

**Table 2:** Weighted characteristics of the study population based on ALI quartiles.

	ALI quartiles				*p*-value
	Q1	Q2	Q3	Q4	
	*N* = 9,867	*N* = 9,867	*N* = 9,867	*N* = 9,868	
Age (years)	50.43 (18.48)	47.13 (16.72)	46.08 (16.07)	45.36 (15.70)	<0.0001
Sex					<0.0001
Male	4,509 (45.70%)	4,614 (46.76%)	5,042 (51.10%)	4,878 (49.43%)	
Female	5,358 (54.30%)	5,253 (53.24%)	4,825 (48.90%)	4,990 (50.57%)	
Race					<0.0001
Mexican American	662 (6.71%)	806 (8.17%)	927 (9.40%)	955 (9.68%)	
Other Hispanic	425 (4.31%)	517 (5.24%)	596 (6.04%)	581 (5.89%)	
Non-Hispanic White	7,386 (74.86%)	7,068 (71.63%)	6,598 (66.87%)	5,712 (57.88%)	
Non-Hispanic Black	631 (6.40%)	728 (7.38%)	1,014 (10.28%)	1926 (19.52%)	
Other Race	762 (7.72%)	748 (7.58%)	731 (7.41%)	694 (7.03%)	
Education Level					0.0001
Below high school	1,693 (17.16%)	1,556 (15.77%)	1,493 (15.13%)	1,683 (17.06%)	
High school	2,368 (24.00%)	2,292 (23.23%)	2,289 (23.20%)	2,358 (23.90%)	
Above high school	5,798 (58.76%)	6,015 (60.96%)	6,072 (61.54%)	5,823 (59.01%)	
Unrecord	8 (0.08%)	4 (0.04%)	13 (0.13%)	2 (0.02%)	
PIR					0.0002
<1.3	2,118 (21.47%)	2029 (20.56%)	2030 (20.57%)	2,140 (21.69%)	
1.3 to <3.5	3,645 (36.94%)	3,460 (35.07%)	3,414 (34.60%)	3,691 (37.40%)	
≥3.5	4,103 (41.58%)	4,379 (44.38%)	4,423 (44.83%)	4,036 (40.90%)	
BMI (kg/m^2^)					<0.0001
<25	4,812 (48.77%)	3,348 (33.93%)	2,217 (22.47%)	1,478 (14.98%)	
25 to <30	3,193 (32.36%)	3,499 (35.46%)	3,466 (35.13%)	2,898 (29.37%)	
≥30	1862 (18.87%)	3,020 (30.61%)	4,184 (42.40%)	5,492 (55.65%)	
Alcohol drinking status					0.2916
Never drinker	1,132 (11.47%)	1,029 (10.43%)	1,055 (10.69%)	1,073 (10.87%)	
Former drinker	1,284 (13.01%)	1,212 (12.28%)	1,295 (13.13%)	1,364 (13.82%)	
Current drinker	7,451 (75.52%)	7,626 (77.29%)	7,517 (76.18%)	7,431 (75.31%)	
Smoking status					<0.0001
Never smoker	2,515 (25.49%)	2,321 (23.52%)	2,494 (25.28%)	2,437 (24.70%)	
Former smoker	4,958 (50.25%)	5,414 (54.86%)	5,458 (55.32%)	5,696 (57.72%)	
Current smoker	2,394 (24.27%)	2,132 (21.61%)	1915 (19.40%)	1735 (17.58%)	
Hypertension					<0.0001
Yes	3,122 (31.64%)	2,931 (29.71%)	2,988 (30.28%)	3,353 (33.98%)	
No	6,731 (68.22%)	6,924 (70.17%)	6,862 (69.55%)	6,507 (65.94%)	
Unrecord	14 (0.14%)	12 (0.12%)	17 (0.17%)	8 (0.09%)	
Diabetes					0.0077
Yes	1,219 (12.35%)	1,016 (10.30%)	1,150 (11.66%)	1,205 (12.21%)	
No	8,648 (87.65%)	8,851 (89.70%)	8,717 (88.34%)	8,663 (87.79%)	
CVD					<0.0001
Yes	1,196 (12.12%)	792 (8.03%)	723 (7.33%)	707 (7.16%)	
No	8,671 (87.88%)	9,075 (91.97%)	9,144 (92.67%)	9,161 (92.84%)	
Cancer					<0.0001
Yes	1,327 (13.45%)	917 (9.29%)	812 (8.23%)	744 (7.54%)	
No	8,531 (86.46%)	8,937 (90.57%)	9,047 (91.69%)	9,112 (92.34%)	
Unrecord	9 (0.09%)	13 (0.14%)	8 (0.08%)	13 (0.13%)	
CKD					<0.0001
Yes	1986 (20.13%)	1,329 (13.47%)	1,199(12.15%)	1,145 (11.60%)	
No	7,881 (79.87%)	8,538 (86.53%)	8,668 (87.85%)	8,723 (88.40%)	
UACR(mg/g)	48.72 (19.84)	32.15 (13.74)	23.67 (8.57)	21.12 (7.41)	<0.0001
eGFR (mL/min/1.73m^2^)	90.62 (24.69)	94.05 (21.50)	95.06 (21.03)	96.51 (20.82)	<0.0001

CKD, chronic kidney disease; PIR, poverty income ratio; BMI, body mass index; CVD, cardiovascular disease; UACR, urinary albumin to creatinine ratio; eGFR, estimated glomerular filtration rate; ALI, advanced lung cancer inflammation index. Continuous variables were described by weighted mean (standard deviation), categorical variables were described by unweighted frequencies (*n*) and weighted percentages (%).

### 3.2 Association between ALI and CKD

The results of the multivariate regression analyses for ALI and CKD are summarized in Table 3. The results showed that in the unadjusted model (Model 1), each 10 unit increase in ALI was associated with an 8% reduction in the likelihood of subjects developing CKD (OR = 0.92, 95% CI: 0.91–0.94, *p* < 0.0001), and in the fully adjusted for potential confounders (Model 3), each 10-unit increase in ALI was associated with a 7% reduction in the likelihood of a subject developing CKD (OR = 0.92, 95% CI: 0.91–0.94, *p* < 0.0001). We further converted ALI from a continuous variable to a categorical variable (quartiles), and in the 3 models, the OR values for the second, third, and fourth ALI quartiles were progressively lower compared with the lowest quartile, suggesting that subjects were relatively less likely to have CKD as the level of ALI increased. All the trends were statistically significant (P for trend <0.05). In the fully adjusted model, each unit increase in ALI in quartile 4 subjects was associated with a 55% reduction in their likelihood of developing CKD compared with quartile 1 (P for trend <0.05). The stratified analysis by BMI (Supplementary Table S1) reveals that a significant negative correlation (*p* < 0.05) between ALI and the prevalence of chronic kidney disease (CKD) persists across different groups. Among participants with a BMI <25, the risk of CKD decreases the most for every 10-unit increase in ALI. When ALI is further converted from a continuous variable to a categorical variable (quartiles), the OR values for the second, third, and fourth ALI quartiles were progressively lower compared with the lowest quartile, consistent with the trend observed in Table 3. Moreover, when BMI is <25 or BMI ≥30, the negative correlation between ALI and CKD was not significant for the second ALI quartile (*p* > 0.05) after full adjustment for potential confounding factors (Model 3).

**Table 3:** Weighted multifactorial logistic regression of ALI and CKD.

	Model 1	Model 2	Model 3
	OR (95%CI) *p*-value	OR (95% CI) *p*-value	OR (95% CI) *p*-value
Continuous variable			
ALI per 10 U	0.92 (0.91, 0.94) <0.0001	0.95 (0.94, 0.96) <0.0001	0.93 (0.91, 0.95) <0.0001
Quartile variable			
Q1 (2.83–44.36)	Reference	Reference	Reference
Q2 (44.37–61.9)	0.62 (0.57, 0.67) <0.0001	0.73 (0.67, 0.80) <0.0001	0.66 (0.56, 0.77) <0.0001
Q3 (61.91–83.40)	0.55 (0.50, 0.61) <0.0001	0.69 (0.62, 0.77) <0.0001	0.62 (0.51, 0.74) <0.0001
Q4 (≥83.41)	0.52 (0.47, 0.58) <0.0001	0.65 (0.58, 0.72) <0.0001	0.55 (0.46, 0.65) <0.0001
P for trend	<0.0001	<0.0001	<0.0001

Model 1, no covariates were adjusted. Model 2, age, sex, and race were adjusted. Model 3, age, sex, race, education level, BMI, drinking status, smoking status, hypertension, diabetes, cardiovascular disease and cancer were adjusted.

OR, odds ratio; CI, confidence interval; ALI, advanced lung cancer inflammation index.

**Figure 2:**
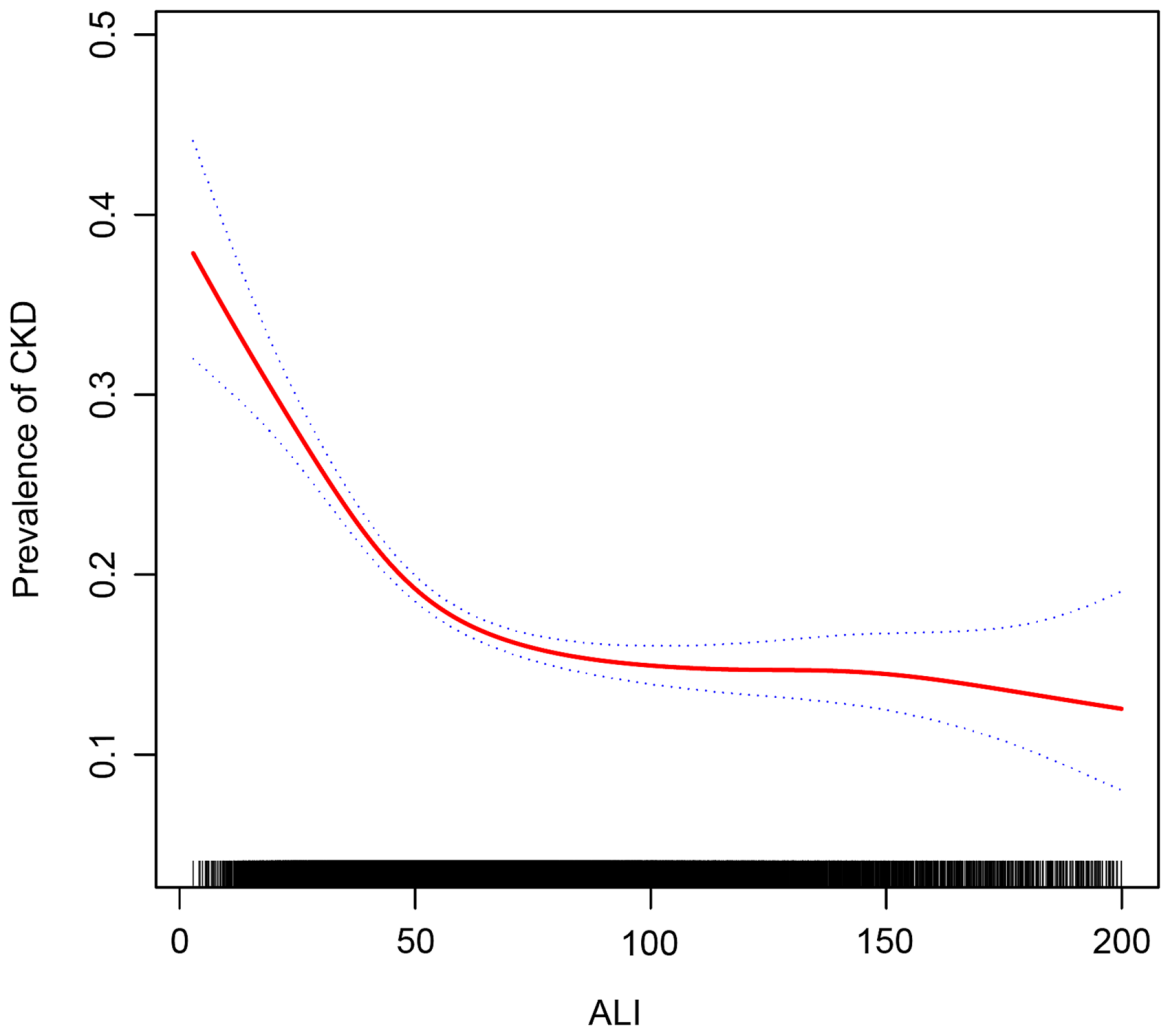
Association between ALI and chronic kidney disease. The red solid line indicates the smooth curve fit between the variables. The blue dashed line indicates the 95% confidence interval of the fit.

We used smoothed curve fitting to explore the potential relationship between ALI and CKD. Data with an ALI greater than 200 will be removed from the curve fit as those data points are considered outliers. The model adjusted for covariates including: age, gender, race, education level, PIR, BMI, drinking status, smoking status, hypertension, diabetes, CVD, and cancer. The results showed that there was an “L-shaped” negative correlation between ALI levels and CKD, with the OR curve for CKD first decreasing sharply with increasing ALI levels and then levelling off after a specific point (Figure 2). Further analysis of the threshold effect showed that ALI had a saturating effect at the 55.09 inflection point, and the negative correlation with CKD was stronger before the ALI reached 55.09, while after the ALI exceeded this inflection point, even if the ALI continued to increase, the effect of the reduction of the risk of CKD was no longer significant in the subjects, and the differences were statistically significant (*p* < 0.05) (Table 4).

**Table 4:** Threshold effects of ALI on CKD analyzed using linear regression models.

	Adjusted OR (95% CI) *p*-value
Fitting by the standard linear model	0.9930 (0.9915, 0.9944) <0.0001
Fitting by the two-piecewise linear model	
ALI	
Inflection point	55.09
ALI < 55.09	0.9790 (0.9748, 0.9831) <0.0001
ALI > 55.09	0.9974 (0.9955, 0.9993) 0.0074
Log likelihood ratio	<0.001

### 3.3 Subgroup analyses

**Figure 3:**
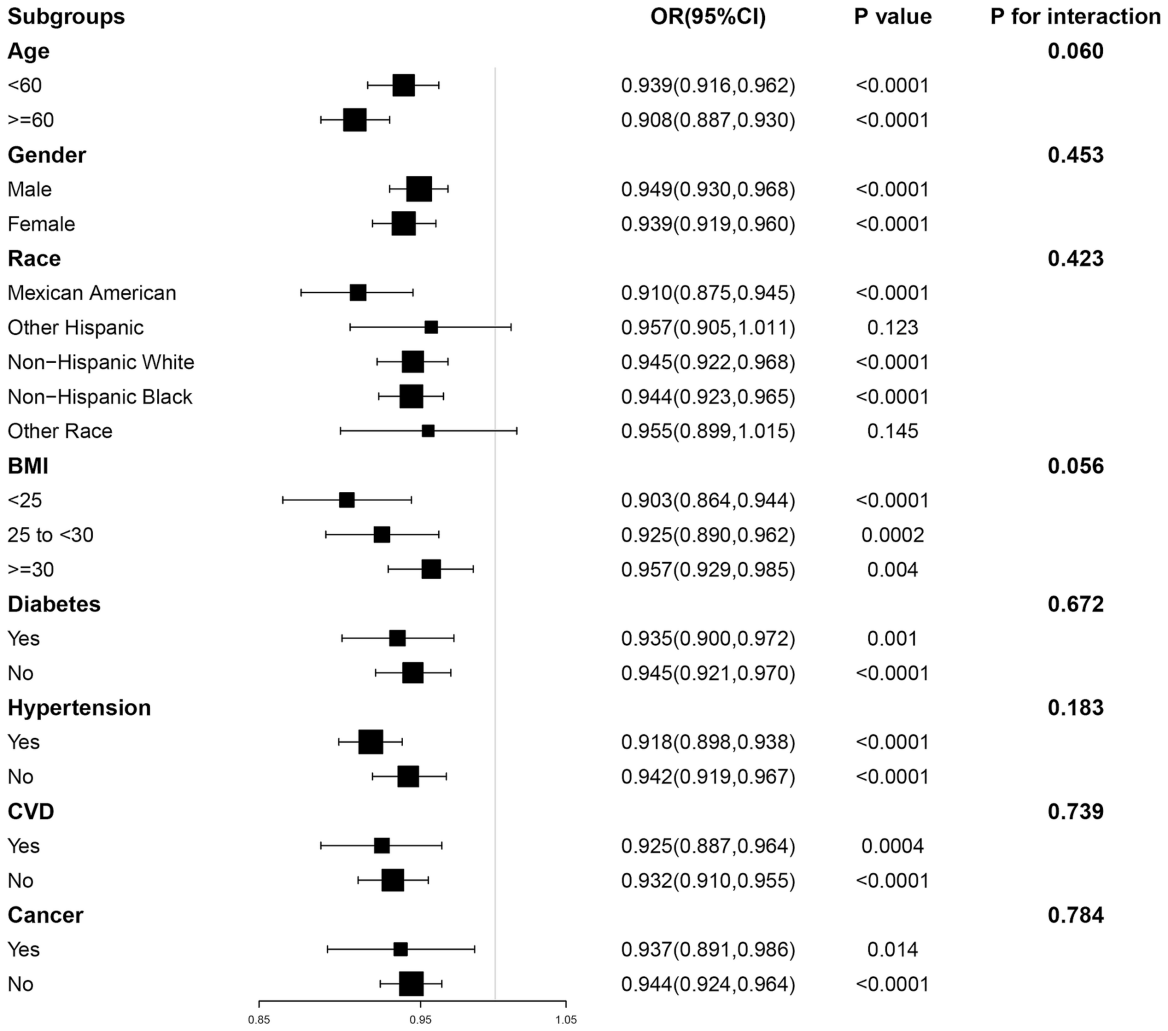
The association between ALI and CKD by different subgroups. BMI, body mass index; CVD, cardiovascular disease; OR, odds ratio; CI, confidence interval.

To assess the stability of our findings and explore whether any specific subgroups manifested unique effects, we further employed subgroup analyses (Figure 3). In subgroups stratified by age, gender, BMI, diabetes, hypertension, CVD and cancer, ALI was significantly negatively associated with CKD (*p* < 0.05). In addition, in the interaction test conducted across subgroups, the relationship between ALI and CKD did not show statistical differences across all subgroups, suggesting that differences between subgroups did not have a significant effect on this negative association (P for interaction >0.05).

## 4 Discussion

This cross-sectional study ultimately included 39,469 participants from the NHANES 2003–2018 cohort for analysis, including 19,121 men and 20,348 women. Of these, 7,204 patients had CKD. ALI levels were lower in patients with CKD compared to the non-CKD population. We found that the higher the ALI of the participants, the less likely they were to have CKD. In addition, the results of smoothed curve fitting and threshold effect analyses showed an L-shaped negative association between ALI levels and CKD, with a saturation effect at the 55.09 inflection point. Subgroup analyses and interaction tests showed that the association was consistent across subgroups. To our knowledge, this study is the first to investigate the association between ALI and CKD.

CKD has a high prevalence and mortality rate and has become a global public health crisis (25). A growing body of evidence suggests that inflammation plays an important role in the progression of CKD due to multiple etiologies (26). Increased production and decreased clearance of pro-inflammatory cytokines, oxidative stress and acidosis combine to promote the development of a chronic inflammatory state in CKD (27, 28). A prospective cohort study showed that serum inflammatory markers hs-CRP, and interleukin-6 (IL-6) were significantly elevated with the progression of CKD (29). In addition, recent studies have found a positive correlation between SII and SIRI, the systemic inflammatory markers based on peripheral blood cells, and CKD (10, 11). All of the above studies suggest that inflammation exerts a detrimental effect in the development of CKD patients, which is consistent with our findings. However, it is worth noting that previous studies usually relied on a single inflammatory marker to predict the risk of developing CKD. Malnutrition is a common complication in patients with CKD, which is characterized by lower serum albumin levels and decreased BMI (30). A cross-sectional study showed that malnutrition was associated with a high risk of developing CKD (31). Another study that included 682 CKD patients found that malnutrition was a risk factor for infectious complications in CKD patients, with a risk 2.41 times higher than that of non-malnourished patients. As chronic kidney disease progresses, malnutrition accelerates the development of infectious complications in CKD patients (32). In addition, patients with CKD are also susceptible to a coexisting state of malnutrition, inflammation, and metabolic disorders (7, 33), making it crucial to identify composite indicators of nutrition and inflammation.

The ALI is calculated by multiplying BMI by albumin and dividing by NLR, and unlike single inflammatory markers, it allows for a comprehensive assessment of systemic status by combining nutritional and inflammatory factors. Previous research has demonstrated that ALI exhibits superior predictive capability in patients with nutritional and inflammation-related conditions. Song et al.’s findings suggest that ALI has the best performance in predicting prognosis in lung cancer patients compared to other inflammatory/nutrition-based measures (16). A retrospective study found that preoperative ALI levels predicted short-term and long-term prognosis in gastric cancer patients undergoing gastrectomy (14). In addition, the validity of ALI has been demonstrated in non-cancer populations. Zhang et al. reported that the long-term risk of all-cause mortality in hypertensive patients decreased with increasing ALI (17). A recent study found a significant negative correlation between ALI levels and all-cause and cardiovascular mortality in patients with type 2 diabetes mellitus (T2DM) (18). There are no studies evaluating the association between ALI and CKD. Our study demonstrated for the first time that higher ALI levels were associated with a reduced risk of developing CKD, with consistent results in Models 1, 2, and 3. In addition, the results of interaction analysis showed that age, gender, BMI, hypertension, diabetes, and CVD had no significant effect on the association between ALI and CKD. This suggests that ALI is an independent protective factor for CKD.

This study shows that there is an L-shaped negative correlation between ALI and CKD. I think the possible biological mechanisms can be analyzed in the following aspects. Firstly, the calculation of ALI includes BMI, albumin level, and NLR, all of which are associated with nutritional and inflammatory status in patients with CKD. BMI is the most commonly used and simplest nutritional assessment method in clinical practice, and based on the magnitude of the value, it can determine whether the patient is malnourished or has protein-energy wasting (34). Kao et al. found that in elderly diabetic patients, there was a negative correlation between BMI and CKD, with lower BMI being associated with an increased risk of developing CKD (35). A prospective study that included 395 patients found that higher BMI was associated with higher survival and better prognosis in patients with non-dialysis dependent CKD (36). Obesity is a risk factor for the development of CKD (37), however, the above study showed that higher BMI was protective and associated with better survival, a phenomenon known as the “obesity paradox” (38). Therefore, we believe that BMI plays an important role in this L-shaped non-linear relationship, and that maintaining a high but appropriate BMI may imply better nutritional status in patients with CKD, which contributes to enhanced immune function and thus reduced inflammation and comorbidities. Secondly, serum albumin, which is synthesized by hepatocytes in the liver and secreted into the bloodstream, may reflect a patient’s nutritional status and the severity of the disease they are suffering from. A large prospective cohort study showed a strong negative correlation between serum albumin levels and CKD risk (39). In addition, Jiang et al. found that participants with serum albumin <51.4 g/L had a significantly lower risk of developing CKD with increasing serum albumin (40). In addition, albumin molecules inhibit the expression of pro-inflammatory factors, such as TNF-*α*, IL-1 and IL-6, thereby protecting tissues from inflammatory damage (41, 42). Finally, NLR is a simple ratio between peripheral blood neutrophil and lymphocyte counts, which can reflect the systemic status of inflammation or immune response (43, 44). Neutrophils with pro-inflammatory effects have increased activation in the blood of CKD patients and participate in systemic inflammation by increasing the production of reactive oxygen species (45, 46). In addition, in an inflammatory state, the body’s immune regulation leads to increased apoptosis of lymphocytes, which in turn exerts an immune effect (47). Previous studies have found that higher NLR is independently associated with a higher risk of CKD (8). In addition, a Meta-analysis that included 10 studies showed that NLR was a valid predictor of all-cause mortality and cardiovascular events in patients with CKD (48). In summary, it is feasible to assess nutritional and inflammation levels in CKD patients using the composite index ALI and to explore the correlation with CKD. In addition, we believe that BMI plays a more critical role in this L-shaped nonlinear relationship. Notably, ALI had a saturation effect at the 55.09 inflection point.

Our findings have potential clinical implications. Lower ALI values typically indicate malnutrition and chronic inflammation, conditions associated with a higher risk of CKD. Clinicians may use this information to identify at-risk individuals and initiate early interventions, such as optimizing nutritional support, managing inflammation, and developing personalized treatment plans, which may slow CKD progression. As a composite index, ALI offers a more holistic assessment of a patient’s nutritional and inflammatory status compared to single markers, which can enhance clinical decision-making and supplement existing risk assessment tools. Additionally, our results align with the concept of the “obesity paradox,” suggesting that maintaining an appropriate BMI may be protective in CKD patients. This highlights the importance of focusing on nutritional management, particularly in CKD patients with a lower BMI, where targeted nutritional interventions could improve survival and quality of life.

However, this study has several limitations. First, because of the cross-sectional study design, we could not directly demonstrate a causal relationship between elevated ALI and reduced CKD risk. Second, in assessing whole-body nutritional status, we chose BMI and ALB as indicators, both of which may have limitations in terms of specificity and sensitivity. In addition, although we adjusted for some confounders, there may still be potential confounders that were not included because neutrophil and lymphocyte counts may be affected by inflammatory factors that were not considered. Finally, because this study is based on NHANES data from the U.S. adult population and excludes minors, further research is required to determine whether the association between ALI and CKD is generalizable to other populations.

## 5 Conclusion

ALI is an objective, simple, and low-cost composite indicator. Our findings reveal a significant correlation between ALI levels and CKD, underscoring its potential utility in monitoring the inflammatory and nutritional status of individuals with CKD. Future prospective studies with larger sample sizes are still needed to confirm this finding.

## Data Availability

The original contributions presented in the study are included in the article/Supplementary material, further inquiries can be directed to the corresponding author.
