# Supplementary material for: Association between advanced lung cancer inflammation index and chronic kidney disease: a cross-sectional study
Source: Front Nutr. 2024 Oct 4;11:1430471. doi: 10.3389/fnut.2024.1430471 (PMC11486730; doi:10.3389/fnut.2024.1430471)
Supplement: Supplementary file 1 [file Data_Sheet_1.PDF]

Supplementary Table 1. Weighted multifactorial logistic regression of ALI and CKD stratified by BMI

BMI (kg/m^2)	Continuous variable<br>ALI per 10 U<br>OR (95%CI)<br>P-value	Quartile variable<br>Q1<br>OR (95%CI)<br>P-value	Q2<br>OR (95%CI)<br>P-value	Q3<br>OR (95%CI)<br>P-value	Q4<br>OR (95%CI)<br>P-value
<25					
Model 1	0.84 (0.81, 0.88)<br><0.0001	Reference	0.53 (0.46, 0.62)<br><0.0001	0.40 (0.33, 0.48)<br><0.0001	0.37 (0.31, 0.46)<br><0.0001
Model 2	0.90 (0.87, 0.93)<br><0.0001	Reference	0.65 (0.55, 0.77)<br><0.0001	0.54 (0.44, 0.67)<br><0.0001	0.52 (0.42, 0.66)<br><0.0001
Model 3	0.89 (0.85, 0.93)<br><0.0001	Reference	0.78 (0.59, 1.02)<br>0.0757	0.51 (0.39, 0.68)<br><0.0001	0.54 (0.40, 0.73)<br>0.0001
25 to <30					
Model 1	0.86 (0.84, 0.89)<br><0.0001	Reference	0.52 (0.45, 0.61)<br><0.0001	0.44 (0.37, 0.52)<br><0.0001	0.38 (0.31, 0.46)<br><0.0001
Model 2	0.91 (0.88, 0.93)<br><0.0001	Reference	0.63 (0.53, 0.74)<br><0.0001	0.58 (0.48, 0.69)<br><0.0001	0.52 (0.43, 0.63)<br><0.0001
Model 3	0.93 (0.89, 0.97)<br>0.0005	Reference	0.66 (0.50, 0.87)<br>0.0042	0.64 (0.49, 0.84)<br>0.0018	0.60 (0.44, 0.81)<br>0.0016
⩾30					
Model 1	0.93 (0.91, 0.94)<br><0.0001	Reference	0.66 (0.57, 0.78)<br><0.0001	0.58 (0.50, 0.69)<br><0.0001	0.54 (0.46, 0.63)<br><0.0001
Model 2	0.94 (0.93, 0.96)<br><0.0001	Reference	0.79 (0.67, 0.93)<br>0.0045	0.71 (0.60, 0.84)<br>0.0002	0.64 (0.54, 0.76)<br><0.0001
Model 3	0.96 (0.93, 0.98)<br>0.0026	Reference	0.87 (0.67, 1.12)<br>0.2756	0.77 (0.60, 0.99)<br>0.0412	0.73 (0.56, 0.94)<br>0.0179

Model 1, no covariates were adjusted. Model 2, age, sex, and race were adjusted. Model 3, age, sex, race, education level, drinking status, smoking status, hypertension, diabetes, cardiovascular

disease and cancer were adjusted.

BMI: body mass index; OR: odds ratio; CI: confidence interval; ALI: advanced lung cancer inflammation index.
